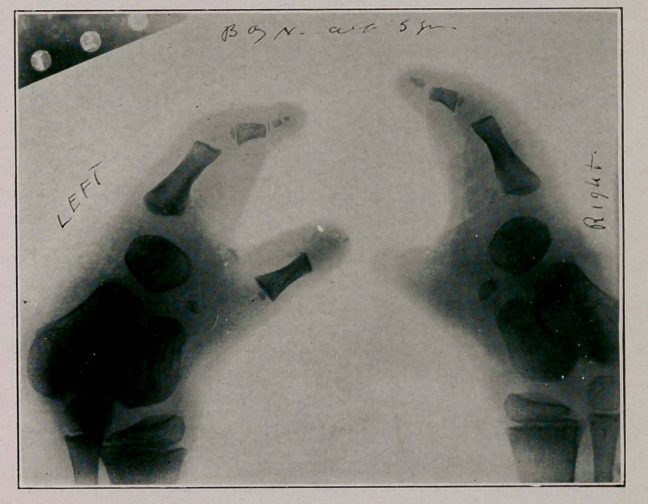# Monodactylism

**Published:** 1914-10

**Authors:** L. G. Hanley

**Affiliations:** Buffalo, N. Y.


### Monodactylism

By L. G. HANLEY, M. D., LL. D.,

Buffalo, N. Y.

Notes of Case: Master H., aged 5, parents healthy, first and only child, no hereditary or other cause apparent. Father 30, mother 26, labor normal, pregnancy normal throughout, no shock, fright or "maternal impression." Boy perfectly formed except for the monodactylism illustrated. Mentally bright and can read, write and draw well for one so young. Can grasp objects and has a strong grip. The finger flexes upon the extremity of the fore arm—one cannot say **wrist** as the carpal bones are absent as well as the tarsal. He walks and runs without difficulty.

## Figures and Tables

**Figure f1:**
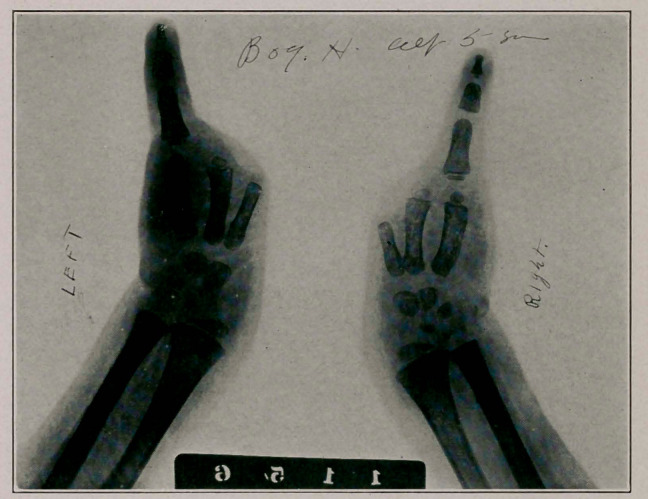


**Figure f2:**